# Editorial: Sustainable production of bioactive compounds within lignocellulosic biorefineries

**DOI:** 10.3389/fbioe.2022.1110908

**Published:** 2023-01-12

**Authors:** Marina Tišma, Mirela Planinić, Jian Hao, Edivaldo Ximenes Ferreira Filho, Maobing Tu

**Affiliations:** ^1^ Faculty of Food Technology, University of Osijek, Osijek, Croatia; ^2^ Shanghai Advanced Research Institute, Chinese Academy of Sciences (CAS), Pudong, China; ^3^ University of Brasilia, Brasilia, Brazil; ^4^ University of Cincinnati, Cincinnati, OH, United States

**Keywords:** sustainable, bioactive compounds, lignocellulose, biorefinery, biomaterials

Lignocellulosic biorefineries play an important role in the development of a circular and sustainable bioeconomy worldwide. Although a wide variety of different value-added products and bioenergy can be derived from lignocellulosic materials, the key Research Topic is to find the methods that are economically sustainable.

In order to gain new knowledge in the field of lignocellulosic biorefineries, especially in terms of sustainable production of bioactive compounds, we have been working on the Research Topic “Sustainable Production of Bioactive Compounds within Lignocellulosic Biorefineries”. However, the Research Topic not only includes bioactive compounds, but also findings on the area of biomaterials.

It includes five articles (one review paper, one mini-review and three original research articles) written by scientists from Brazil, China, Croatia, Czech Republic, Germany, Greece, and Slovenia. We hereby express our sincere gratitude for the efforts and contributions of all those involved in this Research Topic.

Two papers present possibilities to convert brewer’s spent grain and sugarcane bagasse into a variety of bioactive compounds. The first paper (Zeko-Pivač et al.) is a review paper which provides a comprehensive overview of the temporary use of brewer’s grains and future goals for its better exploitation in the production of value-added products, targeting the Central and Eastern European markets. It includes market size and growth rate of selected value-added products, namely lactic acid, ferulic acid; 2,3-butanediol, gibberellic acid, xylitol, citric acid, PHB, natural red pigment, ascorbic acid and cordycepin. The second paper is an original research paper by authors from Chile, which presents information on the production of a wide range of bioproducts (such as *p*-hydroxycinnamates and lignin or lignin-carbohydrate complexes) from sugarcane bagasse enzymatically treated with endoxylanase.

In regards to biomaterials, two papers deal with the production of the new generation of biomaterials. A mini-review paper by scientists from China (Lai et al.) looks at enzymatically catalyzed reactions to produce polymer hydrogels, which are expected to be the next-generation of biomaterials for tissue engineering and regenerative medicine. The possibility of producing elastin-like proteins as renewable biobased high-performance polymers with intriguing mechanical properties was explored in another research paper from scientists from Germany (Haas et al.)

Finally, the fifth paper by authors from Greece (Tsapou et al.) presents pulsed electric field (PEF) as a green extraction technology to enhance the extraction of polyols and propanediols from a glycerol.

In summary, the articles published as part of the Research Topic “Sustainable Production of Bioactive Compounds Within Lignocellulosic Biorefineries” provide good insight into the novel approaches related to the pretreatment and/or production of bioproducts from various residues.

